# Dynamic patterns of postprandial metabolic responses to three dietary challenges

**DOI:** 10.3389/fnut.2022.933526

**Published:** 2022-09-22

**Authors:** Patrick Weinisch, Jarlei Fiamoncini, Daniela Schranner, Johannes Raffler, Thomas Skurk, Manuela J. Rist, Werner Römisch-Margl, Cornelia Prehn, Jerzy Adamski, Hans Hauner, Hannelore Daniel, Karsten Suhre, Gabi Kastenmüller

**Affiliations:** ^1^Institute of Computational Biology, Helmholtz Zentrum München, Neuherberg, Germany; ^2^Food Research Center – FoRC, Department of Food Science and Experimental Nutrition, School of Pharmaceutical Sciences, University of São Paulo, São Paulo, Brazil; ^3^Digital Medicine, University Hospital of Augsburg, Augsburg, Germany; ^4^Core Facility Human Studies, ZIEL Institute for Food and Health, Technical University of Munich, Freising, Germany; ^5^Else Kröner Fresenius Center for Nutritional Medicine, School of Life Sciences, Technical University of Munich, Freising, Germany; ^6^Department of Physiology and Biochemistry of Nutrition, Max Rubner-Institut, Karlsruhe, Germany; ^7^Metabolomics and Proteomics Core, Helmholtz Zentrum München, Neuherberg, Germany; ^8^Institute of Experimental Genetics, Helmholtz Zentrum München, Neuherberg, Germany; ^9^Department of Biochemistry, Yong Loo Lin School of Medicine, National University of Singapore, Singapore, Singapore; ^10^Institute of Biochemistry, Faculty of Medicine, University of Ljubljana, Ljubljana, Slovenia; ^11^Institute for Nutritional Medicine, School of Medicine, Technical University of Munich, Munich, Germany; ^12^Department of Food and Nutrition, Technical University of Munich, Freising, Germany; ^13^Department of Biophysics and Physiology, Weill Cornell Medicine—Qatar, Doha, Qatar

**Keywords:** postprandial metabolism, dietary challenge, time-series data, longitudinal metabolomics, metabolic adaptation, response patterns, nutritional metabolomics

## Abstract

Food intake triggers extensive changes in the blood metabolome. The kinetics of these changes depend on meal composition and on intrinsic, health-related characteristics of each individual, making the assessment of changes in the postprandial metabolome an opportunity to assess someone's metabolic status. To enable the usage of dietary challenges as diagnostic tools, profound knowledge about changes that occur in the postprandial period in healthy individuals is needed. In this study, we characterize the time-resolved changes in plasma levels of 634 metabolites in response to an oral glucose tolerance test (OGTT), an oral lipid tolerance test (OLTT), and a mixed meal (SLD) in healthy young males (*n* = 15). Metabolite levels for samples taken at different time points (20 per individual) during the challenges were available from targeted (132 metabolites) and non-targeted (502 metabolites) metabolomics. Almost half of the profiled metabolites (*n* = 308) showed a significant change in at least one challenge, thereof 111 metabolites responded exclusively to one particular challenge. Examples include azelate, which is linked to ω-oxidation and increased only in OLTT, and a fibrinogen cleavage peptide that has been linked to a higher risk of cardiovascular events in diabetes patients and increased only in OGTT, making its postprandial dynamics a potential target for risk management. A pool of 89 metabolites changed their plasma levels during all three challenges and represents the core postprandial response to food intake regardless of macronutrient composition. We used fuzzy c-means clustering to group these metabolites into eight clusters based on commonalities of their dynamic response patterns, with each cluster following one of four primary response patterns: (i) “decrease-increase” (valley-like) with fatty acids and acylcarnitines indicating the suppression of lipolysis, (ii) “increase-decrease” (mountain-like) including a cluster of conjugated bile acids and the glucose/insulin cluster, (iii) “steady decrease” with metabolites reflecting a carryover from meals prior to the study, and (iv) “mixed” decreasing after the glucose challenge and increasing otherwise. Despite the small number of subjects, the diversity of the challenges and the wealth of metabolomic data make this study an important step toward the characterization of postprandial responses and the identification of markers of metabolic processes regulated by food intake.

## Introduction

People with safe access to food, who enjoy on average three main meals per day, spend almost the total of their wake time in a postprandial state, which is characterized by complex physiological processes within a 4–6 h time frame after food intake (1). In this phase, the excess of nutrients challenges the homeostatic system with the demand for adaptation. The adaptive processes are orchestrated across multiple metabolic pathways (2) and across different organs while ensuring the most appropriate usage of energy substrates. Macronutrient partitioning with (i) uptake of glucose into liver and muscle for glycogen production and (ii) fatty acid uptake into adipose tissue for storage in triglycerides are the main routes of clearing postprandial elevations in blood levels (3). Amino acids delivered by dietary proteins are mainly oxidized because of strictly controlled storage capacities and their disappearance is thus driven by energy production (4). The contribution of further metabolites (e.g., of metabolites that originate from consumed food) to postprandial changes in the human metabolome is well recognized. But the dynamics of these changes and their integration into the overall metabolic regulation in the postprandial phase still lack understanding (5).

In recent years, the interest in postprandial metabolism has been rising, as the dynamic metabolic response during the postprandial phase has been linked to health (6, 7). In particular, the magnitude and timing of postprandial changes in glucose and triglyceride levels, which depend on meal composition, and are highly individual even after an identical meal (8, 9), have been shown to associate with the risk of metabolic diseases, such as diabetes (8), cardiovascular disease (10–12), and liver cirrhosis (13). In these studies, postprandial levels of blood glucose or triglycerides allowed better and more sensitive disease predictions than the fasting levels of these metabolites, suggesting the assessment of individual dynamic postprandial responses as a tool for early detection and personalization of interventions in these diseases.

Each meal drives a complex dynamic switch from a fasting to a postprandial phase with time-dependent changes in the levels of numerous metabolites from different pathways in addition to glucose and triglycerides. Through metabolite profiling in blood using state-of-the-art metabolomics methods, the complex, time-resolved metabolic responses to nutrient intake in the central compartment can be measured (14). In a standardized setting of dietary challenges, these profiles can objectively describe the flexibility and robustness of a person's metabolism when exposed to a specific acute nutrient load. Standardized challenge tests comprise defined proportions of carbohydrates, proteins, lipids, and combinations of all three macronutrients. The oral glucose tolerance test (OGTT) is the most widely used standardized challenge (15, 16). Initially, the OGTT was designed for the diagnosis of diabetes, but recently, it has become the main tool in human nutritional studies to assess individual systemic metabolic responses to glucose intake through metabolomics (15–27). Within the OGTT, participants are given an oral bolus of 75 g of glucose after overnight fasting. The glucocentric response within the postprandial phase is mainly characterized by insulin-dependent phenomena including stimulation of glycolysis, inhibition of lipolysis, ketogenesis, and proteolysis. Recent studies made efforts to also standardize (i) mixed meal challenges for research (28), which are designed to reflect macronutrient compositions of everyday meals and thereby bring responses closer to real-life settings, as well as (ii) oral lipid (fat) tolerance tests (OLTT), which contain high amounts of lipids with a pre-defined lipid composition and are used for assessing postprandial hyperlipidemia as a risk factor for cardiovascular disease (29). While the postprandial response to glucose within an OGTT has been characterized systemically using metabolomics approaches in many studies already, recent studies on the responses to mixed meals (28, 30–36) and OLTTs (37–41) mainly focused on glucose and triglyceride levels only (2, 28, 37, 42).

In this study, we aimed to dissect the complexity of healthy systemic postprandial responses by characterizing similarities and differences in the kinetic changes of plasma metabolites across three different dietary challenges (OGTT, mixed meal, and OLTT). We used time-resolved metabolomics in samples of 15 healthy male individuals undergoing the same challenge protocol to record metabolite levels before and during the postprandial period. Out of 634 profiled metabolites, which cover a broad range of biochemical pathways, we identified those metabolites that were responsive to the intake of each meal. The readout allows for the characterization of core postprandial responses, identifying metabolite changing levels regardless of the macronutrient composition of the test meal, as well as the identification of metabolite changes that were unique to a specific challenge. Using a statistical approach for fuzzy clustering, we classify groups of metabolites into response patterns with a detailed description of their typical kinetic behavior in plasma, facilitating functional dissection of human postprandial metabolism.

## Materials and methods

### Study population

For the work presented here, we reused data from a subsample of the Human Metabolome (HuMet) study, which was conducted at the Human Study Center of the Else-Kröner-Fresenius Center for Nutritional Medicine at the Technical University Munich and has been published first in 2012 (43). For HuMet, fifteen healthy male participants were recruited to be as homogeneous as possible, with an average age of 27.8 ± 2.9 years and normal weight [body mass index (BMI) of 23.1 ± 1.8 kg/m^2^]. Participants did not take any medication, and none of the participants showed any metabolic abnormalities according to standard clinical chemistry. More details about the recruitment criteria and entrance examinations can be found in Krug et al. (43). All participants gave their written informed consent, and the ethical committee of the Technische Universität München approved the study protocol (#2087/08), which was in accordance with the Declaration of Helsinki.

### Dietary challenges

As part of the HuMet study, participants underwent six different metabolic challenges performed in two blocks of 2 days with a wash-out period of 4 weeks in between. In total, samples from 56 different time points were collected. For the analyses presented here, we reused data from the second 2-day block. Over the period of 2 days, participants underwent three different nutritional challenges, with samples collected at 20 different time points: an oral glucose tolerance test (OGTT) and a mixed meal challenge [by ingestion of a standard liquid diet (SLD)] on day 1, and an oral lipid tolerance test (OLTT) on day 2.

Here, we compared the postprandial response from the three challenges from baseline (shortly before dietary intake) up to 240 min postprandially. All challenges were administered under highly controlled conditions in the study center. Before entering the study center for 2 days, participants had the same meal (standard size chicken-based with vegetables) as dinner in the evening.

The oral glucose tolerance test (OGTT) drink consisted of a 300-ml solution with mono- and oligosaccharides, equivalent to 75 g of glucose after enzymatic cleavage (Dextro O.G.T., Roche Diagnostics, Mannheim, Germany). Participants were overnight fasted at OGTT baseline. Plasma samples (EDTA) were taken at nine time points (0, 15, 30, 45, 60, 90, 120, 180, and 240 min).

The standard liquid diet (SLD) consisted of a defined fiber-free formula drink (Fresubin^®^ Energy Drink Chocolate, Fresenius Kabi, Bad Homburg, Germany). The challenge drink was adjusted for each participant providing one-third of the daily energy requirement. The SLD challenge directly followed after the OGTT (4 h OGTT = baseline SLD). Within the SLD challenge, plasma samples were taken at five time points (0, 60, 120, 180, and 240 min).

The oral lipid tolerance test (OLTT) combined one part of a fat emulsion containing predefined long-chain triglycerides (Calogen^®^, Nutricia, Zoetemeer, Netherlands) and two parts of the SLD (see above). The volume of the OLTT challenge drink was adjusted for each participant to provide 35 g fat/m^2^ body surface area. Participants were overnight fasted at OLTT baseline. Plasma samples were taken at seven time points (0, 30, 60, 90, 120, 180, and 240 min).

The detailed drink compositions can be found in Supplementary Table 1.

### Non-targeted metabolomics

EDTA-plasma samples were kept at −80°C until the moment of analysis through the non-targeted mass spectrometry (MS)-based HD4 platform at Metabolon, Inc. (Durham, NC, USA), which has been described in detail previously (44). Briefly, after thawing, recovery standards were added to the samples for quality control purposes. Thereafter, metabolites were extracted with methanol using an automated liquid handling device [MicroLab STAR^®^ system from Hamilton Company (Reno, NV, USA)]. After centrifugation, the resulting extracts were split into four portions for analysis and one for reserve. Extracts were placed briefly on a TurboVap^®^ (Zymark) to remove the organic solvent, stored overnight under nitrogen, and reconstituted in solvents compatible with the analytical methods before each analysis. Metabolite detection and quantification were performed using four different analytical methods: two separate reverse phase (RP)/ultra-high-performance liquid chromatography (UPLC)-MS/MS methods with electrospray ionization (ESI) in positive mode, an RP/UPLC-MS/MS with ESI in negative mode, and a hydrophilic interaction liquid chromatography (HILIC)/UPLC-MS/MS with ESI in negative mode. All methods utilized a Waters ACQUITY UPLC and a Thermo Scientific Q-Exactive high resolution/accurate mass spectrometer interfaced with a heated electrospray ionization (HESI-II) source. The Orbitrap mass analyzer operated at a mass resolution (m/Δm) of 35,000.

As the focus of the HuMet study was on the dynamic changes in metabolite levels within individuals, samples from the same individual and 2-day blocks were put on the same plate. Within plates, the order of samples was randomized. Additionally, aliquots of a reference plasma sample were measured on each plate to monitor the overall performance of the analytical methods and to assess the experimental variation of measurements (in terms of the mean relative standard deviation of abundances per metabolite determined in the reference).

For compound identification, experimental spectra were compared to Metabolon's in-house library entries of purified standards or recurrent entities based on retention time/index (RI; calculated based on the recovery standards), mass to charge ratio (m/z) (match to the library +/– 10 ppm), and MS/MS spectral data (MS/MS forward and reverse scores between the experimental data and authentic standards). The area-under-the-curve was used to quantify peaks.

For this study, we only used data from peaks representing identified metabolites. Thereby, the term “metabolite” refers to all low-weight molecules captured by the analytical approaches described, including exogenous molecules (xenobiotics) and small peptides. In total, measurement of the HuMet plasma samples yielded relative quantification for 595 identified metabolites. These metabolites were assigned to eight chemical classes termed super-pathways (Amino Acids, Carbohydrates, Cofactors and Vitamins, Energy, Lipids, Peptides, Nucleotides, Xenobiotics) and 78 sub-pathways (Supplementary Table 2).

The preprocessing of the metabolomics data was based on the complete HuMet data set comprising samples of 56 time points in total: Raw peak area-under-the-curve values of each metabolite were normalized to account for instrument inter-day tuning differences by dividing the values of each metabolite at each run day by the median of values for the metabolite on this day (i.e., setting the run day medians to one).

### Targeted metabolomics and insulin

Plasma samples had also been analyzed using a targeted, mass spectrometry-based metabolomics approach [Absolute*IDQ* p150 kit from Biocrates Life sciences AG (Innsbruck, Austria)] (45) as previously described in detail in Krug et al. (43). In this study, we used the concentrations of the 132 quantified and quality-controlled metabolites as published previously. The metabolites are listed in Supplementary Table 2 and were manually assigned to the metabolite classes “Amino Acids” (*n* = 14), “Carbohydrates” (*n* = 1), and “Lipids” (*n* = 117).

Plasma concentrations of insulin were measured by enzyme-linked immunosorbent assay (ELISA, K219, Dako, Glostrup, Denmark) and were also available and used in this study.

### Data analysis

#### Data pre-processing

We applied semi-manual data curation to the complete targeted and non-targeted HuMet data sets to identify outliers while considering metabolite fluctuations over time. To this end, we systematically filtered single data points according to the two following criteria: (i) the value of the single data point is beyond four times the standard deviation from the mean of the metabolite, and (ii) the data point was not measured within the first 30 min of a study challenge, where largest fold changes in metabolite levels were expected and observed. A total of 163 data points fitted both criteria and were subsequently inspected manually to find data points (metabolite at time point x), where spikes were only seen for single subjects (compared to all other subjects at time point x). After manual inspection, a total of 92 data points were excluded (i.e., set to missing). In the last step, metabolites with more than 30% missingness were excluded from the analysis (*n* = 93). Missing values of the resulting data frame were imputed per platform using the machine learning algorithm *missForest*, which is implemented in the *missForest* R package (version 1.4). The resulting dataset was log_2_ transformed.

For further analysis in this study, we extracted the data points corresponding to the 20 time points within the three dietary challenges from the complete curated data set.

#### Hypothesis tests

For each challenge, we identified metabolites with a significant time effect on their blood levels using the non-parametric ANOVA-like test implemented in the *ld.f1* function of the *nparLD* R package (version 2.1) as proposed by Noguchi et al. (46). This method allows for testing the null hypothesis of “no time effect” in factorial longitudinal data with skewed distributions (or non-homogenous variances) and a limited number of subjects as underlying our study. The *ld.f1* function provides ANOVA-type statistics for the time effect in a homogeneous group of subjects that is observed repeatedly at s = 1, … t time points, which is analogous to a repeated measures ANOVA statistic with metabolite levels as dependent and time as an independent categorical variable while treating subjects as “random effect.” We performed the test for time effects for each challenge separately and encoded time as a categorical, not a continuous variable to reveal whether or not a metabolite changes in response to the different challenges; the relative quantification of metabolites and non-equidistant sampling of time points in our data does not allow for straightforward (quantitative) estimation of the amount of concentration changes per time. Time effects were considered significant for *p* < 7.9 × 10^−5^ after adjusting for multiple testing (0.05/634 = 0.000079).

We additionally performed a paired *t*-test for each metabolite to identify statistically significant changes between overnight fasting status (OGTT baseline) and the SLD baseline time point (240 min after the OGTT) as a *post-hoc* test for estimating potential carryover effects from the OGTT into the SLD challenge. We applied the same multiple testing corrected threshold as above to judge significance. Results are provided in Supplementary Table 8 and visualized in Supplementary Figure 4.

#### Correlation

Pairwise Pearson correlation analysis based on the log_2_ transformed dataset containing metabolites of the core metabolic response was applied to assess the similarity of temporal patterns of metabolite levels with those of insulin across time points of all dietary challenges in all 15 participants. For analysis, we used the *cor.test* function of the R package *stats* (version 3.6.2).

#### Clustering of metabolite trajectories

To identify groups of metabolites that are related to each other functionally in terms of their postprandial regulation, we clustered metabolites of the core postprandial response (*n* = 89) according to similarities in their temporal profiles over all individuals and all challenges. To this end, we used fuzzy c-means clustering [*mfuzz* function of the *Mfuzz* R package (version 2.50.0)], which is commonly used in time-resolved gene expression analysis and has been proven to be robust against noisy time-resolved data. For the clustering, each metabolite was represented by a 300-dimensional vector corresponding to the z-scored data of the 20 time points across the three dietary challenges measured in the 15 subjects (Note that concatenation of the temporal profiles of subjects facilitates grouping metabolites that are highly co-regulated within each individual independent of differences in the baseline abundances of metabolites between subjects or subject-specific amplitudes of the temporal profiles). The clustering algorithm starts by assigning random cluster centers and calculates the Euclidean distance for each data point (= 300-dimensional vector representing a metabolite) to the cluster centers, as well as the so-called membership scores for each metabolite and cluster. These scores range from 0 (low membership probability) to 1 (high membership probability) and describe the probability of cluster membership per metabolite. Next, the algorithm iteratively (in our case 69 iterations) updates cluster centers and re-calculates distances, minimizing the overall distances of data points to cluster centers weighted by their membership scores. Clustering results are provided in Supplementary Table 4 for the 89 metabolites of the core postprandial response (and Supplementary Table 7 for all metabolites with *p* < 0.05 in all three challenges). For the definition and visualization of temporal patterns of metabolites in **Figure 5**, we assigned each metabolite to the cluster, for which it had the highest cluster membership probability.

Optimal clustering parameters (i.e., fuzzifier *m*, number of clusters *k*) for our data set (i.e., vectors of 89 metabolites with significant postprandial responses) were estimated as proposed by Schwämmle and Jensen (47) [as implemented in *mestimate* and *cselection* functions of the *Mfuzz* R package (version 2.50.0)]. As a result, the fuzzifier was set to *m* =1.25, and the optimal number of clusters was set to *k* = 8.

## Results

**Figure 1:**
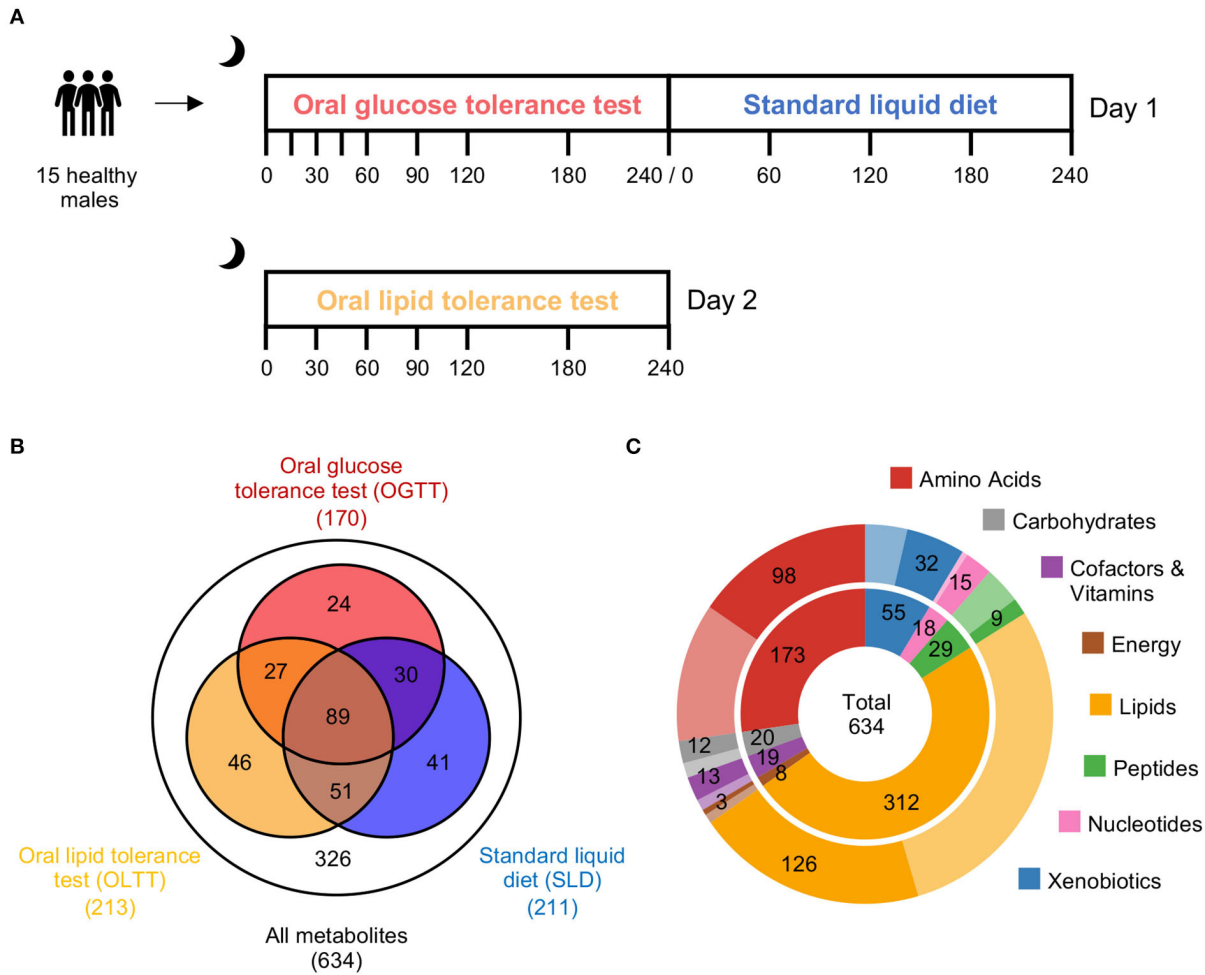
Metabolic responses to three dietary challenges. **(A)** Fifteen young healthy male participants completed three dietary challenges. Up to eight blood plasma samples were taken from baseline to 4 h postprandially in intervals from 15 to 60 min. Each sample was analyzed using a non-targeted and a targeted mass spectrometry-based metabolomics approach to identify metabolites increasing or decreasing in response to a challenge in a time-resolved manner. **(B)** Out of 634 measured metabolites that survived data quality control, we identified those metabolites in each challenge that displayed a significant change during the 4 h after ingestion of the challenge drink (based on a repeated measure ANOVA-type statistics; Bonferroni adjusted *p* < 0.05). The Venn diagram illustrates the number of overlapping and unique metabolites with significant responses to the challenges with 89 metabolites that showed changes irrespective of the composition of the meal. **(C)** The 308 metabolites with significant postprandial responses are distributed over the 8 investigated metabolite classes (saturated color in the outer circle indicates the portion of significant metabolites in the respective class in relation to the number of metabolites measured from this class, which is shown in the inner circle).

In this study, we contrasted and compared the dynamic metabolic responses to three standardized dietary challenges with different macronutrient compositions in a homogenous group of 15 healthy male participants. Specifically, participants completed an oral glucose tolerance test (OGTT), a standard liquid diet (SLD) challenge, and an oral lipid tolerance test (OLTT) with up to eight blood samples drawn across a span of 4 h following ingestion of the challenge drink (Figure 1A). Time-resolved profiling of plasma samples yielded the circulating levels of 634 metabolites measured on two metabolomics platforms, including a non-targeted platform with 502 metabolites and a targeted platform with 132 metabolites after quality control. The resulting data matrix thus contained 634 metabolites × 20 sampling time points × 15 participants.

### Responses of the plasma metabolome to different dietary challenges

First, we analyzed the fluctuations of metabolite blood levels during the 4-h period in each of the three challenges separately. To identify metabolites whose levels change in response to a challenge, we used robust non-parametric ANOVA-type statistics that allows testing for time effects in a small group of homogeneous subjects that are observed repeatedly at 1, … t time points [*t* = 9 (OGTT), 5 (SLD), 7 (OLTT)]. Metabolites with a significant change (Bonferroni adjusted *p* < 0.05) across the sampling time points were considered to have a postprandial response. In total, 308 out of 634 metabolites showed a postprandial response in at least one of the dietary challenges (Supplementary Table 2). These metabolites are spread over all eight investigated metabolite classes [Amino Acids (*n* = 98), Carbohydrates (*n* = 12), Cofactors and Vitamins (*n* = 13), Energy (*n* = 3), Lipids (*n* = 126), Nucleotides (*n* = 9), Peptides (*n* = 15), and Xenobiotics (*n* = 32); Figure 1C]. Out of the 308 postprandially altered metabolites, 89 metabolites showed significant changes in all three challenges (Figure 1B). Twenty-four metabolites displayed changes only during the OGTT, 41 were specific to the SLD challenge, and 46 were specific to the OLTT.

Increases or decreases of circulating metabolites following dietary intake reflect the dynamics of postprandial metabolism and the content of the ingested meal. Most of the 170 metabolites that changed in response to the glucose challenge in the OGTT were decreased from the time of ingestion (baseline) to 1 h postprandially and the further course of the test until 4 h postprandially. In the OLTT and the SLD challenge, more metabolites were increased during the course of the challenges.

**Figure 2:**
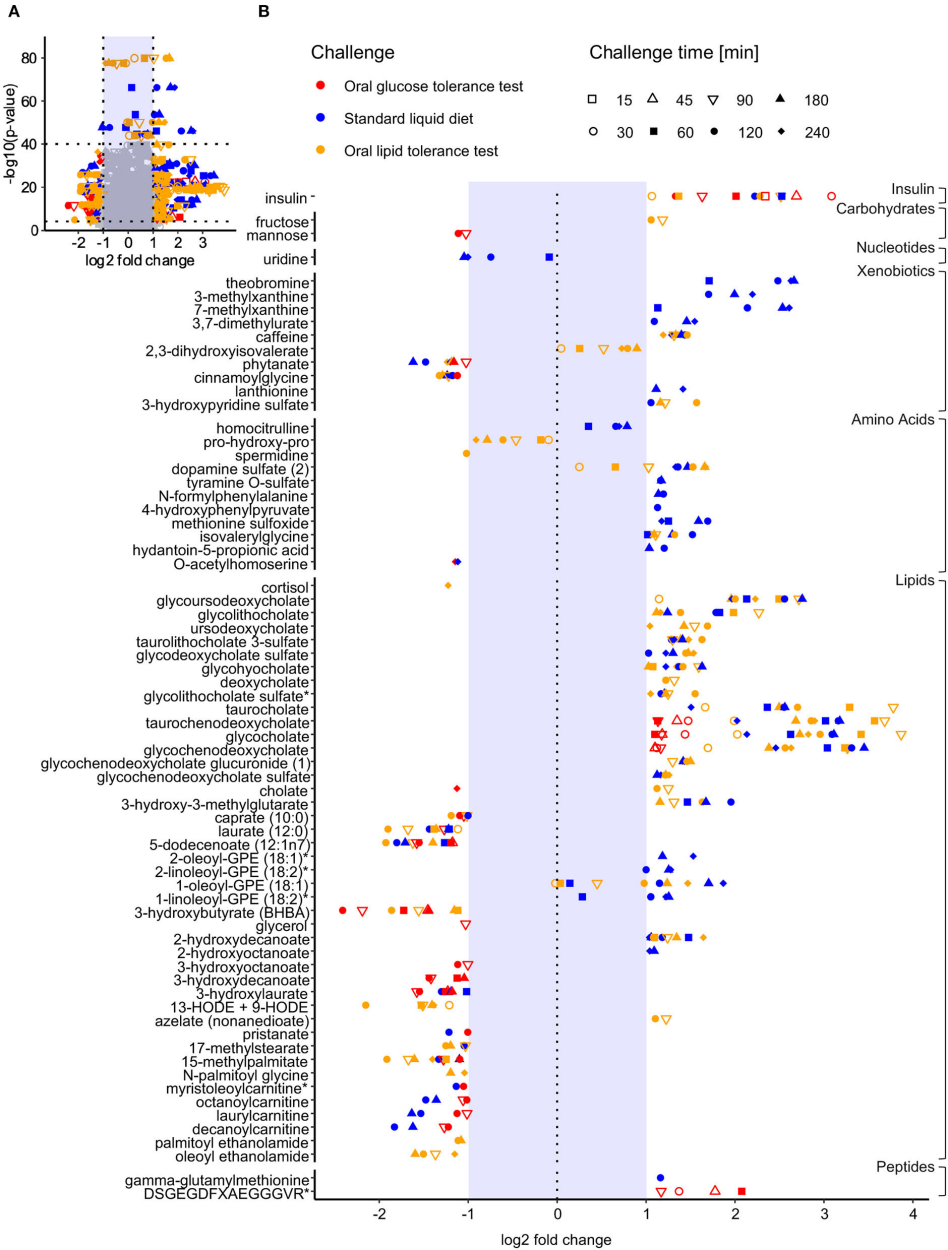
Fold changes of metabolites at different sampling time points after ingestion for results with largest effects/smallest *p*-values. **(A)** Volcano plot showing the log_2_ fold change of each metabolite in each challenge after each sampled time point (time points are indicated by different symbols) in relation to the *p*-value (ANOVA-type statistic) that the metabolite reached in the test for significant postprandial changes in a challenge, i.e., each metabolite is displayed multiple times per challenge. For each challenge, only metabolites with any significant postprandial change and large fold changes [abs(log_2_fc) > 1] or with very low *p*-values [–log_10_(*p*-value) > 40] are colored (red: OGTT; blue: SLD; yellow: OLTT) in this plot. **(B)** Metabolites/time points meeting one of both coloring criteria in the volcano plot are integrated into the forest plot, which displays the observed log_2_ fold changes. The gray plot band depicts the band of log_2_fc between −1 and 1, for which the fold changes are shown only if the *p*-value of the metabolite was very low for a particular challenge (i.e., if the *p*-value of the metabolite meets the above threshold). Otherwise, fold changes with abs(log_2_fc) < 1 are not displayed.

**Figure 3:**
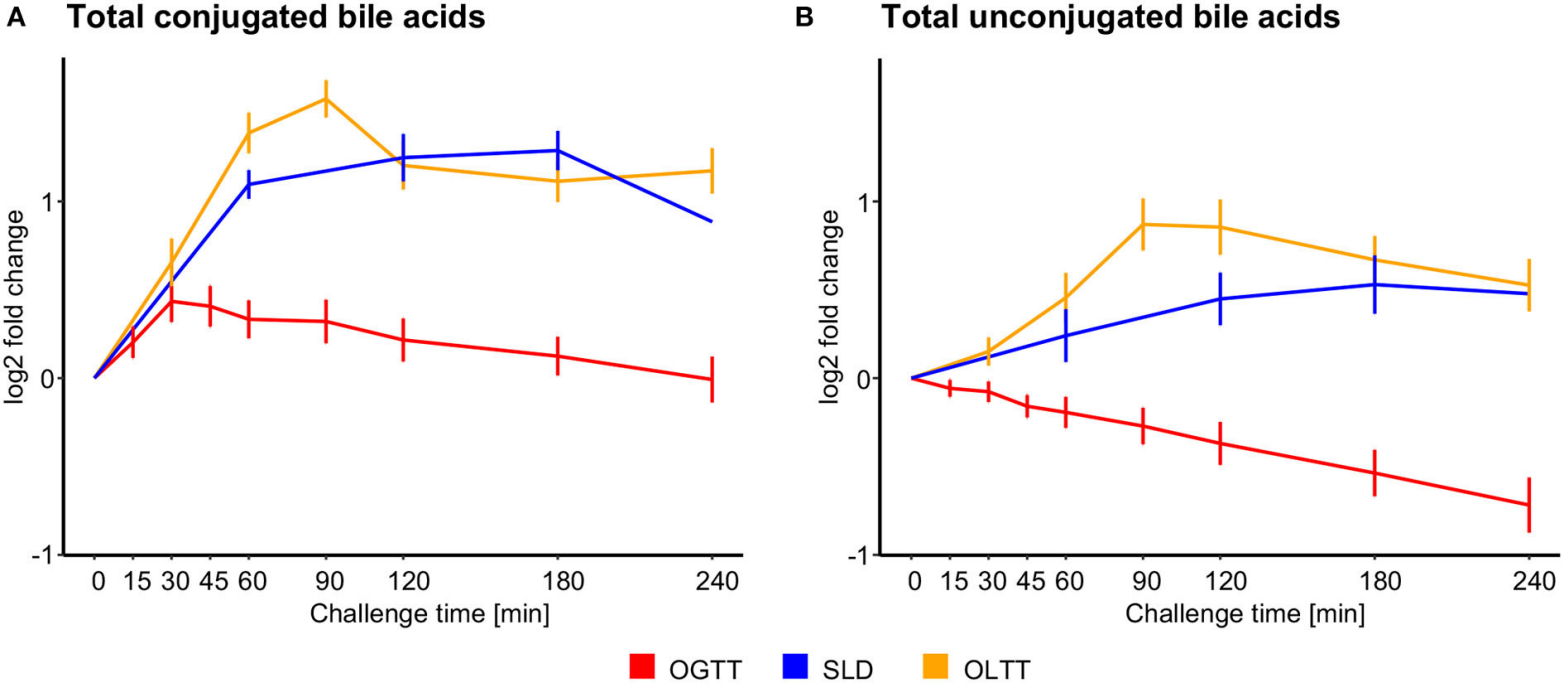
Temporal plots of sums of conjugated and unconjugated bile acids. Bile acids that exhibited significant temporal changes in at least one of the challenges were split into unconjugated (*n* = 6) and conjugated (*n* = 3) compounds. For each group, we calculated the total sum of abundances (based on normalized ion counts). **(A)** Log_2_ fold changes in the sum of conjugated bile acids ± the standard error of the mean (SEM) after the ingestion of the different challenge drinks. **(B)** Log_2_ fold changes in the sum of unconjugated bile acids ± SEM.

Conjugated bile acids showed the largest postprandial increases observed in our study with log_2_ fold changes of 3.87, 3.78, and 3.68 after 90 min in the OLTT for glycocholate, taurocholate, and taurochenodeoxycholate, respectively, and log2 fold changes of 3.45, 3.18, 3.11, and 2.75 for glycochenodeoxycholate, taurochenodeoxycholate, glycocholate, and glycoursodeoxycholate after 180 min in the SLD challenge, respectively (Figure 2, Supplementary Table 3). Glycocholate, glycochenodeoxycholate, and taurochenodeoxycholate levels more than doubled (log_2_ fold change > 1) also in response to the glucose challenge after 30 min in the OGTT. In contrast, for the measured unconjugated bile acids cholate, deoxycholate, and ursodeoxycholate and their sum, we observed much smaller increases in the OLTT and SLD challenge, and decreasing levels in the OGTT (Figure 3).

Metabolites with the strongest decreases involved mainly lipids, including the ketone body 3-hydroxybutyrate (log_2_fc = −2.41) after 120 min in the OGTT, the oxylipin hydroxyoctadecadienoic acids [13-HODE + 9 HODE; log_2_fc = −2.15)], and the median-chain fatty acids 5-dodecaenoate (12:1) (log_2_fc = −1.92) and laurate (12:0) (log_2_fc = −1.9) after 120 min in the OLTT, and decanoylcarnitine (log_2_fc = −1.82) after 120 min in the SLD challenge (Figure 2, Supplementary Table 3). In response to the SLD, the xenobiotic phytanate also showed a large decrease (log_2_fc = −1.61).

While we observed large average fold changes for the metabolites mentioned above, we saw a high variance in the postprandial responses across our 15 healthy male participants for some of these metabolites. For example, for the bile acids glycocholate, glycochenodeoxycholate, and taurochenodeoxycholate, subject 11 showed log_2_ fold changes of 6.39, 4.64, and 5.54 at 90 min in the OLTT, respectively, while these bile acids did not increase to the same extent in subject 2 with log_2_ fold changes in these three bile acids being only 1.72, 1.90, and 2.10, respectively (Supplementary Figure 1). In contrast, in our analysis, we also found metabolites with relatively small fold changes but very consistent postprandial alterations across the participants (i.e., with low inter-individual variance in fold changes) resulting in low *p*-values. Examples include increases in 2,3-dihydroxyisovalerate (log_2_fc_max_ = 0.90, *p* = 1.03 × 10^−44^ at 180 min) and dopamine sulfate levels (log_2_fc_max_ = 1.67, *p* = 2.77 × 10^−80^ at 240 min) in the OLTT, decreases in levels of the dipeptide prolyl-hydroxyproline (pro-hydroxy-pro) in the OLTT (log_2_fc_min_ = −0.91, *p* = 1.32 × 10^−78^ at 240 min), increases in homocitrulline (log_2_fc_max_ = 0.79, *p* = 2.69 × 10^−45^ at 180 min) and decreases in uridine (log_2_fc_min_ = −1.05, *p* = 1.88 × 10^−48^ at 180 min) levels in the SLD challenge. Also, the glycerophospholipids 1-oleoyl-GPE (18:1) and 2-linoleoyl-GPE (18:2) showed low inter-individual variance in fold changes resulting in the smallest *p*-values for postprandial changes in the SLD challenge.

### Characterization of the core postprandial response and related dynamic patterns

**Figure 4:**
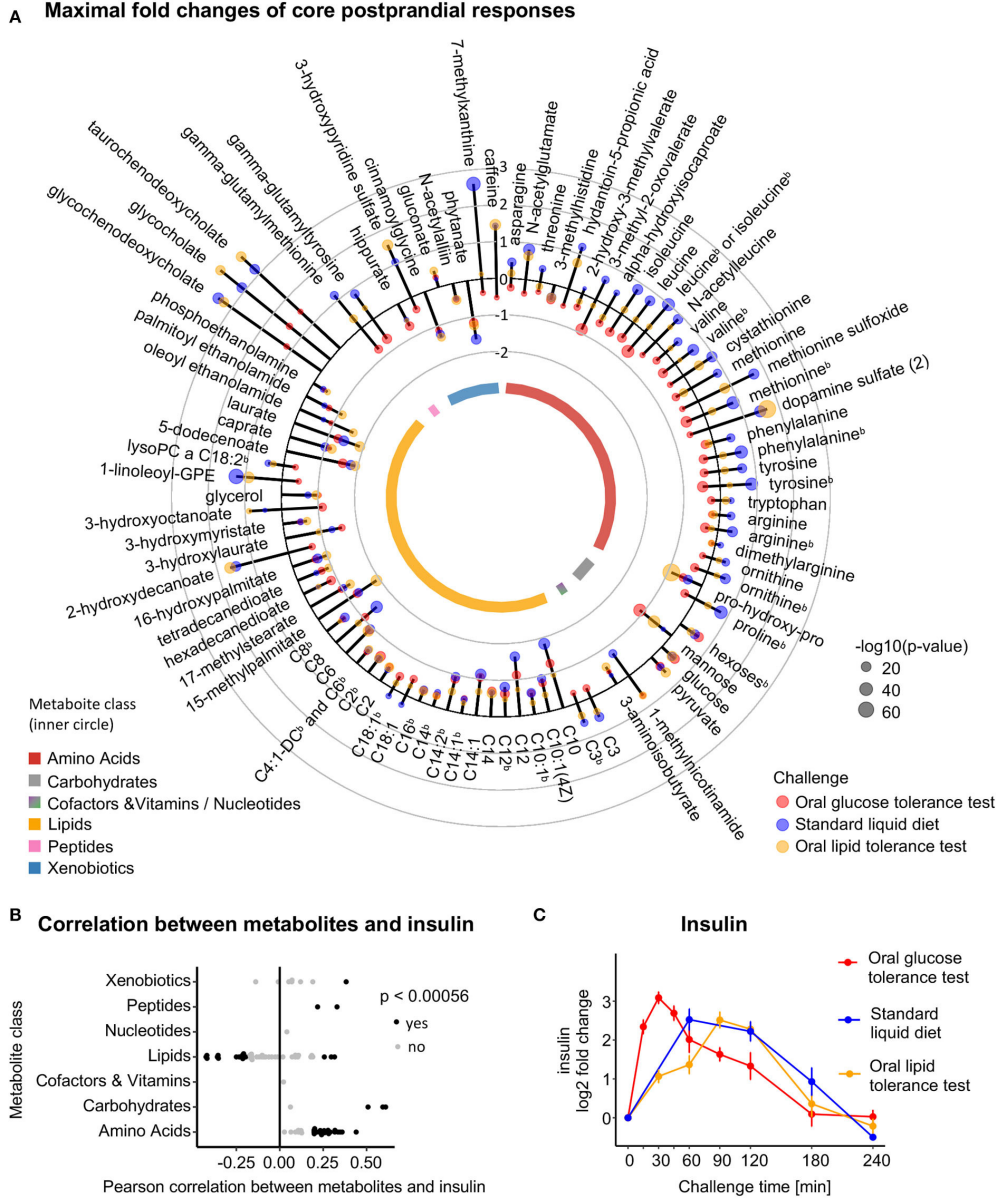
Metabolite pool of the core postprandial response to dietary intake. **(A)** Circular plot of the largest absolute log_2_ fold changes of the 89 metabolites that display significant concentration changes in all three challenges. Lollipop length represents the largest log_2_ fold change in a metabolite within each challenge; colors represent the challenge and the size of the bubble represents the –log10(*p*-value); the gray circles indicate log_2_ fold changes from −2 to 3; ^b^indicates metabolites measured using targeted metabolomics (Absolute*IDQ* p150). **(B)** Pearson correlation of the 89 core metabolites with insulin sorted by metabolite class and colored by significance after multiple testing (black: Bonferroni adjusted *p* < 0.05). **(C)** Time-resolved log_2_ fold changes of insulin levels in relation to the time of ingestion (*t* = 0) for the three challenges (red: OGTT; blue: SLD challenge; yellow: OLTT). Data are represented as mean over the 15 participants ± SEM.

A total of 89 metabolites showed significant postprandial changes in each dietary challenge in our study regardless of the macronutrient composition of the corresponding challenge drinks. We, therefore, refer to this set of metabolites as the metabolite pool of the core postprandial response (Figures 1B, 4A, Supplementary Table 2). The responsive metabolites are distributed across seven metabolite classes, namely, Amino Acids (*n* = 31), Carbohydrates (*n* = 4), Cofactors and Vitamins (*n* = 1), Lipids (*n* = 42), Nucleotides (*n* = 1), Peptides (*n* = 2), and Xenobiotics (*n* = 8). While all of these metabolites show significant changes after ingestion of any of the challenge drinks, the responses were not necessarily in the same direction across the three challenges (Figure 4). Focusing on the peak changes, we observed consistent directions for all carbohydrates, most of them increasing consistently (↑3, ↓1), and for the majority of lipids (↑3, ↓31, ↑↓ 8), most of them decreasing consistently, in the postprandial phase. In particular, acylcarnitines consistently decreased after ingestion of the challenge drinks with the exception of propionylcarnitine (C3) and octadecenoylcarnitine (C18:1), which increased in the OLTT and the SLD challenge. As described already in the previous section, bile acids are consistently increasing in all three dietary challenges. In contrast, we observed postprandial blood level changes in different directions for most amino acids and their derivatives [Amino Acids (↓2, ↑↓ 29); Peptides (↑↓ 2)]: while most of these metabolites increased in the OLTT and the SLD challenge, they significantly decreased in the OGTT. 3-methylhistidine and pro-hydroxy-pro, which showed significant decreases in all three challenges, were the only exceptions.

With insulin being one of the main driving hormones in postprandial regulation, we analyzed the proportion of metabolites that are presumably linked to insulin regulation and follow a similar or mirror-like pattern of temporal postprandial changes as insulin (Figure 4C). To this end, we calculated the Pearson correlation between the time-resolved levels of each measured metabolite and those of insulin across the three challenges and all participants. After adjusting for multiple testing of 89 metabolites, 45 metabolites showed a significant Pearson correlation with insulin (Figure 4B, Supplementary Table 6).

**Figure 5:**
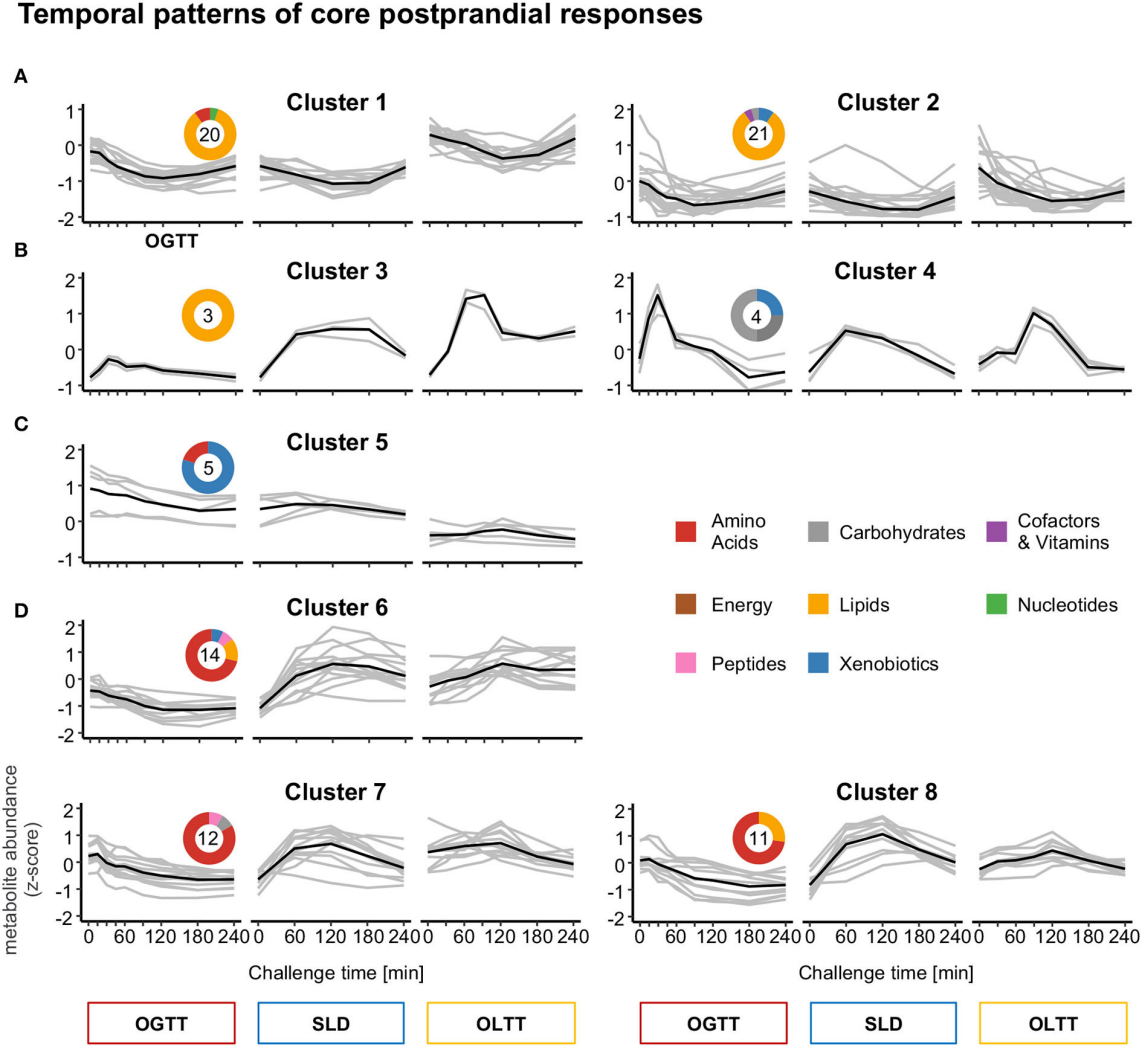
Temporal patterns of core postprandial responses identified by fuzzy c-means cluster analysis. Graphs show four general patterns of responses **(A–D)**. The black line depicts the mean z-score of trajectories over the 15 participants for each metabolite in a cluster. The colored donuts depict the distribution of metabolites in a cluster over the eight metabolite classes. **(A)** Metabolic responses (Cluster 1, 2) with postprandial decreases and increases from baseline until 4 h. **(B)** Responses with postprandial increases and decreases (Cluster 3, 4) from baseline until 4 h. **(C)** Response with steady decreasing trajectories (Cluster 5) from OGTT to OLTT. **(D)** Dissimilar metabolic responses in OGTT compared to SLD/OLTT (Cluster 6–8).

The majority of the 89 metabolites whose levels change in each challenge display non-linear variations over the 4 h of the observed postprandial phase, i.e., levels are not steadily decreasing or increasing but show more complex temporal patterns with, for example, a maximum or minimum before concentrations return to levels similar to those before ingestion. To characterize the observed patterns, and to group metabolites showing similar patterns, we performed clustering of the individual temporal profiles of metabolite levels (z-scores) across all three challenges (20 time points in total) and all 15 participants using the fuzzy c-means algorithm. This approach yielded eight metabolite clusters, which can be further grouped into four general types of curve trajectories (Figures 5A–D, Table 1):

**Table 1:** Clusters of metabolites with similar dynamic patterns within the metabolite pool of the core postprandial response.

**Cluster**	**Pattern**	**Classes**	**Metabolites and insulin**	
1	A	Lipids (17) Amino acids (2) Nucleotides (1)	2-hydroxy-3-methylvalerate 3-aminoisobutyrate 3-hydroxylaurate 3-hydroxyoctanoate alpha-hydroxyisocaproate C10:1 (decenoylcarnitine)^a^ C12 (dodecanoylcarnitine)^a^ C14:1 (tetradecenoylcarnitine)^a^ C14:2 (tetradecadienylcarnitine)^a^ C4:1-DC (fumarylcarnitine) and C6 (hexanoylcarnitine)^a^	C8 (octanoylcarnitine)^a^ C18:1 (octadecenoylcarnitine)^a^ cis-4-decenoyl carnitine, Decanoylcarnitine Hexanoylcarnitine Laurylcarnitine Myristoleoylcarnitine, Myristoylcarnitine Octanoylcarnitine Oleoylcarnitine
2	A	Lipids (17) Xenobiotics (2) Cofactors and Vitamins (2) Carbohydrates (1)	1-methylnicotinamide 15-methylpalmitate 16-hydroxypalmitate 17-methylstearate 3-hydroxymyristate 5-dodecenoate (12:1n7), Acetylcarnitine, C14 (tetradecanoylcarnitine)^a^ C16 (hexadecanoylcarnitine)^a^ C2 (acetylcarnitine)^a^ Caprate (10:0)	Cinnamoylglycine Glycerol Hexadecanedioate Laurate (12:0) Mannose Oleoyl ethanolamide Palmitoyl ethanolamide, Phosphoethanolamine Phytanate Tetradecanedioate
3	B	Lipids (3)	Glycochenodeoxycholate, Glycocholate	Taurochenodeoxycholate
4	B	Carbohydrates (2) Xenobiotics (1) + Insulin	Gluconate Glucose	H1 (hexose)^a^ Insulin
5	C	Xenobiotics (4) Amino acids (1)	3-hydroxypyridine sulfate 3-methylhistidine Caffeine	Hippurate N-acetylalliin
6	D	Amino acids (10) Lipids (2) Xenobiotics (1) Peptides (1)	1-linoleoyl-GPE (18:2) 2-hydroxydecanoate 3-methyl-2-oxovalerate 7-methylxanthine Dopamine sulfate (2) Gamma-glutamyltyrosine Hydantoin-5-propionic acid	Isoleucine Leucine Methionine sulfoxide N-acetylglutamate N-acetylleucine, tyrosine Valine
7	D	Amino acids (10) Peptides (1) Carbohydrates (1)	Arginine Asparagine Cystathionine Dimethylarginine (SDMA + ADMA) Gamma-glutamylmethionine Methionine	Ornithine Phenylalanine Pro-hydroxy-pro Pyruvate Threonine Tryptophan
8	D	Amino acids (8) Lipids (3)	Arginine^a^ C3 (propionylcarnitine)^a^ Leucine or isoleucine^a^ lysoPC a C18:2^a^ Methionine^a^ Ornithine^a^	Phenylalanine^a^ Proline^a^ Propionylcarnitine Tyrosine^a^ Valine^a^

^a^indicates metabolites measured using targeted metabolomics.

*Pattern A* describes clusters of metabolites with decreases in plasma levels following dietary intake and increases toward baseline levels after 4 h in all dietary challenges (Clusters 1 & 2). Cluster 1 comprises mainly medium- and long-chain acylcarnitines (*n* = 15) and hydroxylated fatty acids (*n* = 4), whose plasma levels all decrease until 2–3 h after meal ingestion before they rise again until the end of observation time after 4 h. Cluster 2 shows a similar pattern, but the decreases in its metabolites are smaller (i.e., with lower amplitude) (Figure 5A). The cluster also consists of acylcarnitines (*n* = 4), and further fatty acid (FA) derivatives [FA (*n* = 3), hydroxylated FA (*n* = 2), methylated FA (*n* = 2), dicarboxylic FA (*n* = 2), FA ethanolamides (*n* = 2)] as well as mannose, glycerol, phosphoethanolamine, 1-methylnicotinamide, and two xenobiotics.

*Pattern B* describes clusters of metabolites with increases following dietary intake in all dietary challenges and decreases toward baseline levels afterward (Figure 5B, Clusters 3 & 4). Cluster 3 consists of the three conjugated primary bile acids glycocholate, glycochenodeoxycholate, and taurochenodeoxycholate. The levels of these metabolites reach their maximal concentration at ~30 min in the OGTT, 90–120 min in the SLD challenge, and 90 min in the OLTT. Increases are largest in response to the lipid challenge and smallest after glucose ingestion. Cluster 4 contains the metabolites that clustered with insulin, namely, glucose (non-targeted MS)/hexoses (targeted MS) and gluconate, showing the largest increases after 30, 60, and 90 min in the OGTT, SLD challenge, and OLTT, respectively. The highest amplitude was observed in the OGTT.

*Pattern C* represented by Cluster 5 comprises metabolites with steady decreases in plasma levels from the first analyzed time point until the last point without showing perturbation by the dietary challenges (Figure 5C). All metabolites in this cluster have previously been found associated with habitual food intake or have even been suggested as dietary biomarkers for the intake of meat (3-methylhistidine) (48), coffee (caffeine, hippurate, 3-hydroxypyridine sulfate) (49–51), and garlic (N-acetylalliin) (51), none of which was contained in the challenge drinks.

*Pattern D* describes clusters of metabolites that show a subtle increase followed by a more or less steady decrease in levels during the OGTT, while plasma levels increase in the SLD challenge and in the OLTT (Figure 5D, Clusters 6–8). The majority of metabolites in clusters of Pattern D are amino acids and related metabolites [e.g., N-acetyl amino acids, propionylcarnitine (C3), dipeptides, dopamine sulfate], with some notable exceptions, including the lipid lysophosphatidylcholine C18:2 in Cluster 8, pyruvate in Cluster 7, and the lipids 1-linoleoyl-GPE (18:2) and 2-hydroxydecanoate, as well as the xenobiotics 7-methylxanthine in Cluster 6.

### Challenge-specific postprandial responses

**Figure 6:**
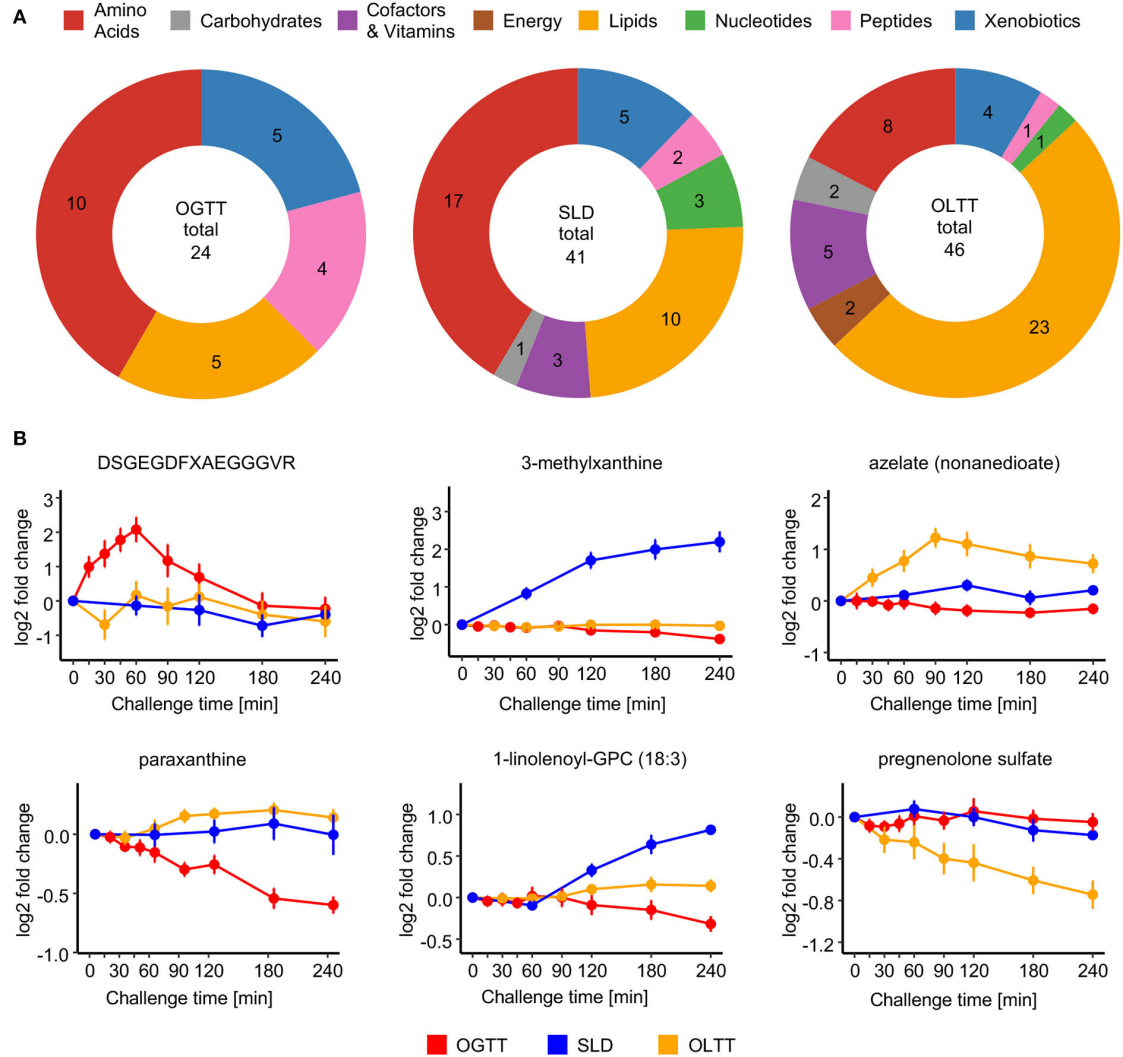
Challenge-specific postprandial responses. **(A)** Distribution of metabolites with significant challenge-specific postprandial responses (Bonferroni adjusted *p* < 0.05) over metabolite classes. **(B)** Temporal profiles for selected examples of metabolites showing challenge-specific postprandial responses. Data are represented as mean across all 15 participants ± SEM.

Next, we focus on metabolites with responses that are only observed in one of the three challenges, i.e., metabolites that are not part of the core metabolic response but depend on the macronutrient composition (or on a specific context). We found 24 compounds responding solely to the glucose stimulus in the OGTT, 41 solely to the SLD challenge, and 46 solely to the lipid-rich drink in the OLTT (Figure 6, Supplementary Tables 2, 5).

For example, the fibrinogen cleavage peptide DSGEGDFXAEGGGVR showed a significant postprandial increase that is specific to the OGTT with a large fold change (log_2_fc = 2.08) at 60 min after glucose ingestion. Other examples of metabolites with OGTT-specific changes in our study are two xenobiotics paraxanthine and theophylline, which are typically associated with coffee consumption (52). While the blood levels of these xenobiotics decreased significantly over the course of the OGTT, which was the challenge test performed first in our study, they were not changed significantly in the SLD challenge or the OLTT, which were performed later, i.e., longer after any prior exposure.

For SLD-specific postprandial changes, examples include several lysoglycerophospholipids, such as 1-linolenoyl-GPC (18:3) and 1-arachidonoyl-GPE (20:4n6), whose blood levels increased prominently in the SLD at some point after ingestion of the drink until the end of the observation after 4 h [1-linolenoyl-GPC (18:3), log_2_fc = 0.84 at SLD 240 min]. Also, the xenobiotic 3-methylxanthine increases exclusively in the course of the SLD (log_2_fc = 2.19 at SLD 240 min).

Most metabolites uniquely significant in the OLTT belong to the Lipids class, including the dicarboxylic acid azelate (non-anedioate), showing OLTT-specific increases up to 90 min after the ingestion of the lipid-rich challenge drink (log_2_fc = 1.22 at OLTT 90 min), and the hormone metabolite pregnenolone sulfate, which steadily decreased over the course of the challenge until 4 h after the ingestion of the lipid-rich challenge drink (log_2_fc = −0.74 at OLTT 240 min).

## Discussion

Our analysis compared the dynamic changes in the metabolite profiles registered during an OGTT, a mixed meal challenge using a standardized liquid diet (SLD), and an OLTT in young healthy participants. The three meal challenges studied here were part of the HuMet study, first presented by Krug et al. (43), for which additional metabolomics data have become available recently (53).

Within the set of 634 analyzed metabolites, we identified a pool of 89 metabolites representing the core postprandial response to food intake regardless of the macronutrient composition of the corresponding challenge drinks. Grouping these metabolites according to similarities in their dynamic response patterns across the three challenges allowed us to dissect the observable human postprandial metabolism into the different insulin-regulated (and seemingly insulin-independent) processes while pinpointing challenge-specific differences in their kinetics.

In addition, we identified metabolites that showed strong postprandial responses that were specific for a particular challenge test, such as increases in the plasma levels of a fibrinogen cleavage peptide observed only in the OGTT, and the increase in the dicarboxylated compound azelate observed only in the OLTT.

A complex meal not only provides macro- and micronutrients that appear in blood but also elicits a hormone response, dominated by insulin and glucagon. While glucagon is considered a hormone released in periods of hunger, insulin drives most of the postprandial changes in metabolite levels. Insulin is thus the most important determinant in homeostatic regulation, affecting the metabolism of sugars, amino acids, and lipids (54). In case of the standardized OGTT, the effects of glucose ingestion on the complete metabolome have been well-studied (15–18, 20, 55, 56). An SLD as used in our study representing a mixed diet has only seldomly been studied (28, 34), and likewise also the OLTT represents a mixed high-fat/high-energy meal (57–61).

While the plain administration of glucose in the OGTT provides a robust insulin-driven change in the blood, the complex mixture of nutrients provided *via* the SLD and OLTT complicates the analysis of postprandial processes as metabolite changes due to nutrients entering the circulation can overlay metabolite changes due to postprandial regulatory processes. Moreover, mixed diet challenge tests and lipid tolerance tests used in studies so far are less standardized than OGTT, further complicating the comparison of results.

What makes our approach novel is that we used extremely well-defined enteral nutrition solutions that can be employed as reference material without any variation in composition by natural or seasonal variation of foods and ingredients. Through the HuMet study, time-resolved metabolomics data are available for the OGTT, and these standardized mixed meal and lipid challenge tests were performed on the same participants. Based on these data, we were able to directly compare the metabolome-wide postprandial responses across these different dietary challenges. We thereby complement recent insights into the dynamic postprandial changes of single metabolites, such as glucose or triglycerides, after challenges with different macronutrient compositions (8, 9) (and the inter-individual variation of these responses).

The core metabolite pool for the postprandial responses across the three challenges is represented by 89 metabolites. For a major fraction of these metabolites, the dynamic changes in levels are insulin-dependent (confirmed by significant correlations with the (temporal) profiles of insulin across the 15 study participants). Insulin dynamics in the OGTT shows maximal levels at 30 min, which is delayed to 60 min in the SLD and to 90 min in the OLTT. This is likely an effect of the higher caloric density provided by the SLD and OLTT and in particular by the higher fat content that causes a reduced gastric emptying to accommodate the nutrient load in the upper small intestine to the capacity for digestion and absorption (62, 63).

Similar differences in shape and peak time of metabolite response curves between challenges are also apparent for most of the other metabolites in the core metabolite pool (Figure 5). For example, the three conjugated primary bile acids glycocholate, glycochenodeoxycholate, and taurochenodeoxycholate significantly increased after ingestion of the lipid-containing challenge drinks in the SLD and OLTT and also after glucose ingestion in the OGTT, as reported in other OGTT challenge studies (15, 16, 18, 56); they form Cluster 3, following an “increase-decrease” (mountain-like) pattern in all challenges same as glucose and insulin (Pattern B); however, the response curves, in particular, peak times and heights of these bile acids largely differ between challenges, with levels reaching their maximum at 30 min in the OGTT and at 90 min in the OLTT. Bile acids enter the gut by stimulation of gallbladder contraction induced by the peptide hormone cholecystokinin (CCK), which is released by ingestion of lipids, mixed meals, and glucose (56, 64) followed by a rapid uptake of bile acids in the upper small intestine leading to an appearance in plasma as fast as glucose in the OGTT. Interestingly, unconjugated bile acids were not part of the core response in our study as they did not change significantly in the OGTT.

Metabolite changes documenting the insulin-induced inhibition of lipolysis and blockade of the release of free fatty acids from stores involve mainly fatty acids and acylcarnitines in our study. These metabolites form Clusters 1 and 2 follow a “decrease-increase” (valley-like) pattern (Pattern A). Here, we see less differences in shapes and peak times of the response curves than for metabolites of Pattern B discussed above: in all three challenges, most fatty acids and acylcarnitines decrease for about 2 h with a return to baseline thereafter, reflecting the switch from fatty acid β-oxidation to glycolysis following the glucose load (15–17, 34, 55). In parallel to the reversed utilization of fatty acids as substrates for oxidation, reduced production of ketone bodies becomes visible by β-hydroxybutyrate levels with a 2- to 4-fold decrease when compared with levels after the overnight fast in the OGTT and OLTT (Figure 2).

Insulin inhibits proteolysis and reverses the release of amino acids to an uptake *via* insulin-dependent amino acid transport systems in various tissues (16, 55). In our study, this effect becomes visible in the OGTT with major reductions in levels of branched-chain and aromatic amino acids. In the SLD and OLTT, blood levels of amino acids increase as the corresponding challenge drinks contain proteins. Metabolites of Clusters 6–8 follow this pattern (Pattern D) and consist mainly of amino acids.

One group of metabolites (Cluster 5) displayed a steady subtle decrease over the 4 h postprandial period within each challenge and also from the first challenge (OGTT) to the last performed challenge (OLTT) defining Pattern C. Thus, these changes are seemingly not affected by any of the challenges. Cross-checking these metabolites against online resources, including *FooDB* (65) and *Exposome Explorer* (66), identified these metabolites as compounds derived from natural foods as part of the standardized meal that the volunteers received at 7 p.m. the day before the challenge studies that started at 8 a.m. Volunteers received a ready-to-consume meal (originally frozen) comprised of chicken with vegetables and spices, such as onion and garlic. And, in essence, these ingredients could all be identified in blood by characteristic metabolites either absorbed from the meal or produced in metabolism from corresponding precursors. Examples are 3-methylhistidine (48, 67) derived from carnosine or anserine of the chicken meat and N-acetylalliin (68) derived from onion and garlic. Other such entities are hippurate (49) linked to benzoate metabolism, microbiota, and consumption of plant-based food. However, hippurate in plasma may increase in postprandial plasma when commercial OGTT solutions containing benzoate as a preservative are used (69). Taken together, these observations argue that there is a significant carryover of food-derived entities into the fasting plasma metabolome from food consumed the day before. In this respect, recording of food intake the day before collecting fasting blood early morning as done in most cohort studies may be recommended to be able to define metabolites as food-derived and not as intrinsic or even as disease-related.

In addition to the identification and characterization of the core postprandial responses, the comparison of metabolite changes across the three different challenges allowed us to also highlight changes that are unique for a specific challenge. For example, azelate (nonanedioate, a dicarboxylic acid) only increased significantly in the OLTT. This metabolite is produced by ω-oxidation but apparently only when all other pathways of fatty acid utilization are saturated with the surplus of dietary fat provided in the OLTT (70).

In the OGTT, we observed a large increase in the levels of DSGEGDFXAEGGGVR, which is a derivative of fibrinogen peptide A. This peptide is cleaved from fibrinogen during conversion to fibrin (71) as part of the blood coagulation process. While no change in DSGEGDFXAEGGGVR is seen in the SLD or OLTT, the levels of this peptide are increased 4-fold at 60 min after glucose ingestion. Interestingly, various studies have reported an association of high fasting blood levels of fibrinogen with type 2 diabetes and retinopathy in epidemiological cohorts (72). Also, elevated fibrinogen blood levels have been suggested as an important factor for the increased risk of diabetic patients for cardiovascular events, including stroke and thrombosis (73). Performing different clamp experiments, Stegenga and colleagues could show that hyperglycemia triggers fibrin synthesis and stimulates coagulation irrespective of insulin levels; hyperinsulinemia, on the other hand, impairs fibrinolysis (74). Notably, we saw a huge variation in the log_2_ fold changes of fibrinogen in response to glucose intake between our healthy participants (similar to inter-individual variations observed for bile acid changes). Taken together, this evidence hint at a link between increasing fibrinogen levels in an OGTT and cardiovascular outcomes. While our findings on fibrinogen in OGTT and its relationship to cardiovascular health still need to be replicated and confirmed in larger cohorts, our data suggest that testing the fold change of fibrinogen of an individual during an OGTT in addition to glucose levels might be valuable to better estimate the risk of cardiovascular events in diabetic and non-diabetic patients.

While the reuse of existing data from the HuMet study allowed for characterizing and comparing the dynamic patterns of postprandial responses to different highly standardized nutritional challenges, the specific design of the experiments also comes with several limitations. First, the three compared challenges were performed in sequential order. As a consequence, carryover effects from one challenge to the next cannot be fully excluded, in particular, the distinction whether an observed effect in the SLD is caused by the mixed meal, is a late response to the glucose challenge, or is coming from chronobiological fluctuation is difficult to prove based on the design as both challenges were performed on day 1. For the purpose of the comparison across challenges, a crossover study design would have been more appropriate. Nonetheless, it appears that only a relatively small number of metabolites did not get back to overnight fasting levels 4 h after OGTT (Supplementary Table 8, Supplementary Figure 4). Also, despite the potential carryover, our results for the different challenges resemble observations from previous studies (Supplementary Table 9). Moreover, due to the sequential order of challenges, we were also able to pinpoint carryover effects from prior exposures that might be misinterpreted as challenge responses in studies that only investigate one challenge. A second issue related to the data reuse is that sampling time points and number of sampled time points differ between the investigated challenges as HuMet was not specifically designed for the direct comparison of these three challenges. This leads to restrictions in the type of statistical approach that can be used. Moreover, we might have missed SLD effects that only occur in the first 30 min after meal ingestion.

Besides the study design, the small sample size (only 15 subjects) and lack of diversity (homogenous group of lean, young, males of European ancestry) limit the reliability and transferability of our results to females, as well as older age groups and other ethnicities. While focusing on a homogenous group might have enabled findings that would have been masked by high variation in the postprandial dynamics in a less homogenous group, our results clearly need replication in larger and more diverse cohorts. Future larger studies would also enable us to consider the influence of the gut microbial composition on the postprandial response by sampling and analyzing fecal samples, which was not possible in our study. Finally, most metabolites analyzed in our study were measured using a non-targeted metabolomics approach. This type of analytical approach covers a broad range of metabolic pathways but yields only relative quantifications for the metabolite abundances, which puts limitations on the comparability of fold changes derived in our study with those of future studies.

In summary, we here compared and characterized the dynamic postprandial responses of highly standardized glucose, mixed meal, and lipid challenges in healthy participants in a metabolome-wide fashion. We identified a pool of 89 metabolites capturing the core postprandial response regardless of the macronutrient compositions of challenge meals and dissected this core response into groups with distinct kinetic behaviors. Moreover, our comparison highlighted postprandial responses that are unique for a particular challenge. We believe that our results provide the research community with a valuable reference of metabolites and metabolic pathways that show a postprandial response to an oral glucose tolerance test, as well as a standardizable mixed meal and an oral lipid tolerance test.

## Data availability statement

The datasets for this study can be found in the Download section of the HuMet Repository: http://humet.org (postprandial_non_imputed, postprandial_imputed). Data from the non-targeted metabolomics platform is also available at the MetaboLights Database: http://www.ebi.ac.uk/metabolights (MTBLS89); data from the targeted metabolomics platform is also available at: http://metabolomics.helmholtz-muenchen.de/humet.

## Ethics statement

The studies involving human participants were reviewed and approved by Ethical Committee of the Technische Universität München (#2087/08). The patients/participants provided their written informed consent to participate in this study.

## Author contributions

Conception and design of work: PW and GK. Data collection: MR, TS, WR-M, CP, JA, HH, HD, and KS. Data analysis: PW and JR. Data interpretation: PW, JF, DS, HD, and GK. Drafting the article: PW, JF, and GK. All authors contributed to the critical revision of the article and approval of the final manuscript.

## Funding

GK received funding (through her institution) from the National Institutes of Health/National Institute on Aging through grants RF1AG057452, RF1AG058942, RF1AG059093, U01AG061359, and U19AG063744. KS is supported by the Biomedical Research Program at Weill Cornell Medicine in Qatar, a program funded by the Qatar Foundation.

## Conflict of interest

The authors declare that the research was conducted in the absence of any commercial or financial relationships that could be construed as a potential conflict of interest.

## Publisher's note

All claims expressed in this article are solely those of the authors and do not necessarily represent those of their affiliated organizations, or those of the publisher, the editors and the reviewers. Any product that may be evaluated in this article, or claim that may be made by its manufacturer, is not guaranteed or endorsed by the publisher.
