# Supplementary material for: Dynamic patterns of postprandial metabolic responses to three dietary challenges
Source: Front Nutr. 2022 Sep 22;9:933526. doi: 10.3389/fnut.2022.933526 (PMC9540193; doi:10.3389/fnut.2022.933526)
Supplement: Supplementary file 1 [file Data_Sheet_1.pdf]

Supplementary Material

1 Supplementary Tables

Supplementary Table 1. Dietary composition of administered challenge drinks.

Supplementary Table 2. List of all 634 metabolites after quality control (and insulin) with annotations, P-values for the time effect, maximum log2 fold changes within a challenge, and time at the maximum log2 fold change.

Supplementary Table 3. Metabolites (and insulin) with significant time effect that meet one of two criteria: (i) absolute log2 fold change > 1 or -log10(P-value) > 40.

Supplementary Table 4. Results of fuzzy c-means clustering of metabolites of the core postprandial response (including cluster membership scores).

Supplementary Table 5. Metabolites uniquely significant in only one challenge.

Supplementary Table 6. Correlation of insulin and metabolites of the core postprandial response.

Supplementary Table 7. Results of fuzzy c-means clustering of metabolites with P-value < 0.05 in all three challenges.

Supplementary Table 8. Differences between challenge baselines for estimation of carry-over effects.

Supplementary Table 9. Postprandial responses found in literature for metabolites of the core postprandial response identified in this study

2 Supplementary Figures

Supplementary Figure 1. Heatmap of inter-individual (between-subject) variation of metabolites displayed in Figure 2.

Supplementary Figure 2. Pearson correlation of all identified metabolites (634) with insulin.

Supplementary Figure 3. Dynamic metabolic patterns in fuzzy c-means cluster analysis based on 222 metabolites with $P < 0.05$.

Supplementary Figure 4. Volcano plot displaying differences between challenge baselines.

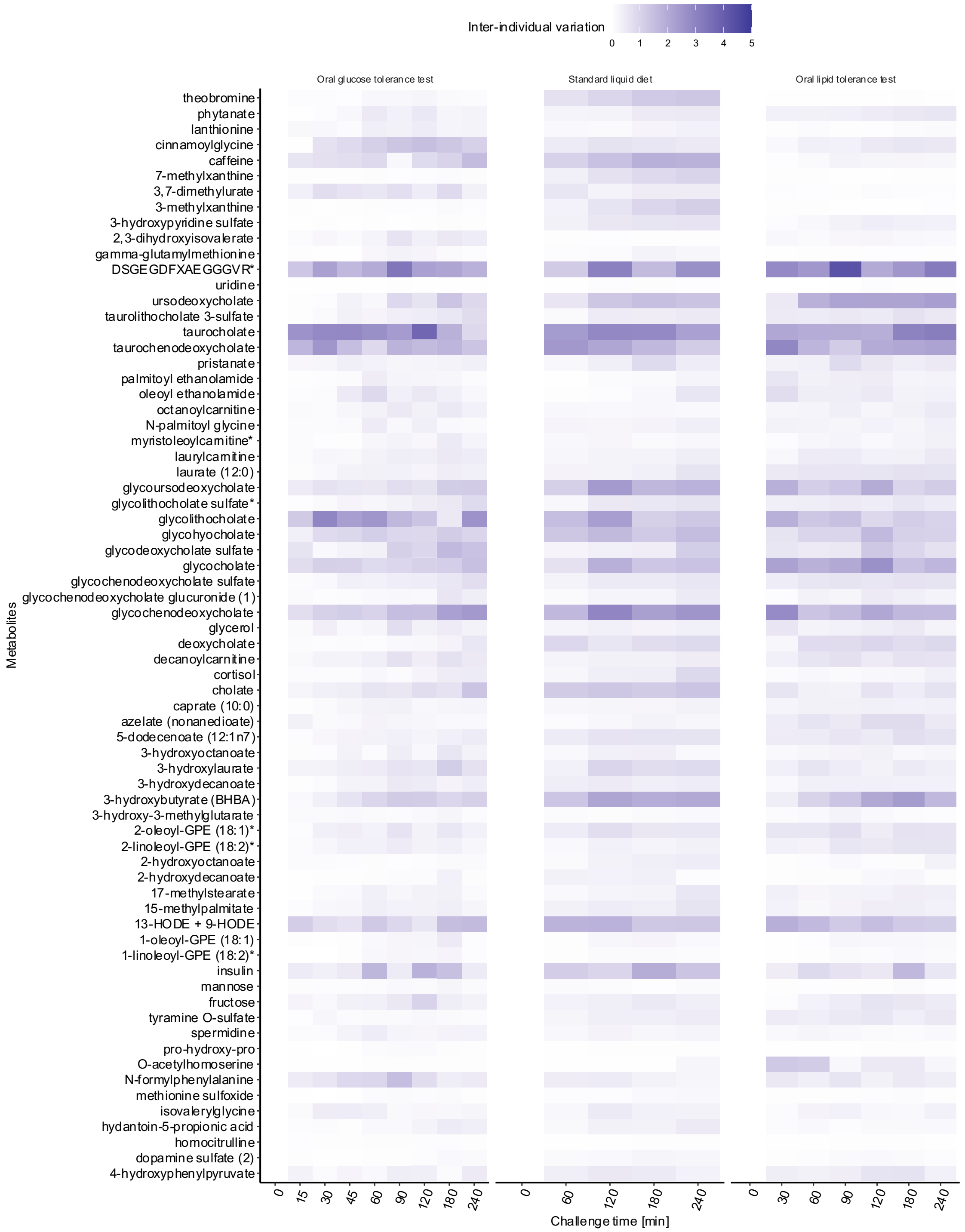

Supplementary Figure 1. Heatmap of inter-individual (between-subject) variation of metabolites displayed in Figure 2. The variance was calculated based on the log2 fold change (difference between t = 0 and respective time point) across all individuals. Only metabolites/time points selected in Figure 2B are displayed, i.e., metabolites/time points following the criteria: metabolite with a significant fold change and (i) an abs(log2fc) > 1 or (ii) -log10(P-value) > 40.

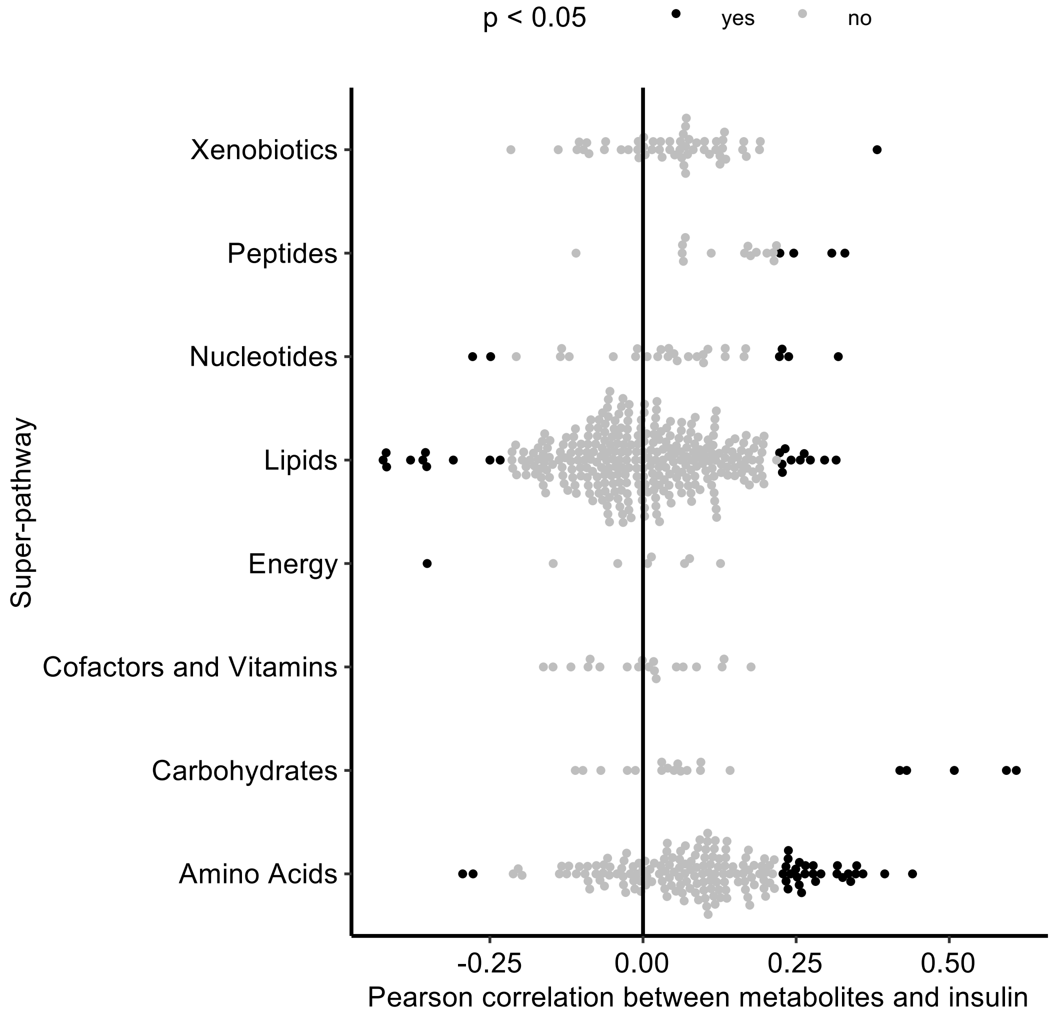

Supplementary Figure 2. Pearson correlation of all identified metabolites (634) with insulin. Pearson correlations were calculated between all 634 metabolites and insulin. Each dot represents a metabolite, which are grouped by metabolite super pathways. The color represents the level of significance (black: Bonferroni adjusted P-value < 0.05). A total of 67 metabolites showed significant correlation evaluated by the *cor.test* function implemented within the *stats* R package (version 4.0.4).

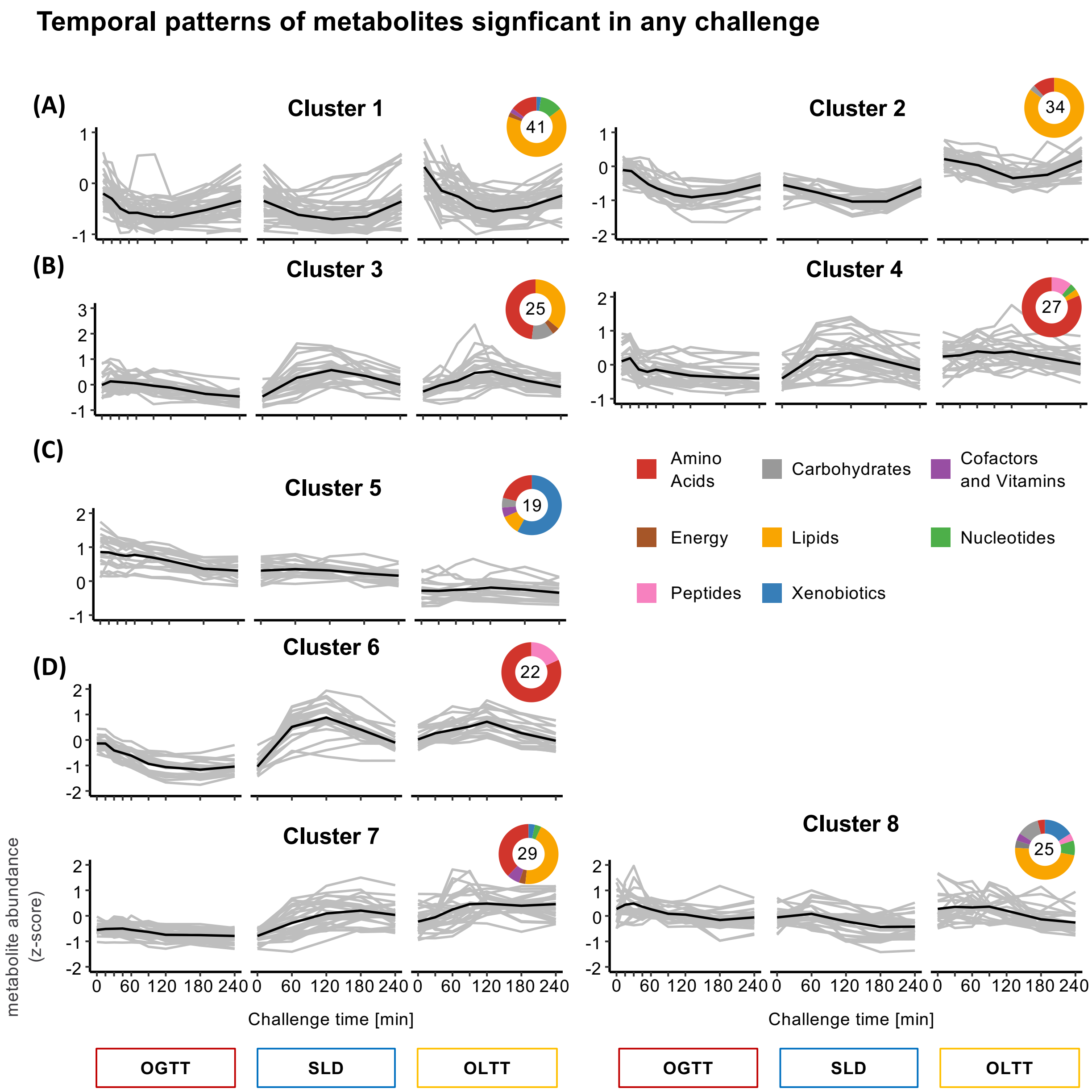

Supplementary Figure 3. Temporal patterns of core postprandial responses identified by fuzzy c-means cluster analysis based on 222 metabolites with P-value < 0.05. Analogous to Figure 5, the graphs show different patterns of responses for the 222 metabolites: (**A**) Metabolic responses (Cluster 1, 2) with postprandial decreases and increases from baseline until four hours. (**B**) Responses with postprandial increases and decreases (Cluster 3, 4) from baseline until four hours. (**C**) Response with steady decreasing trajectories (Cluster 5) from OGTT to OLTT. (**D**) Dissimilar metabolic responses in OGTT compared to SLD/OLTT (Cluster 6-8). The black line depicts the mean z-score of trajectories over the 15 participants for each metabolite in a cluster. The colored donuts depict the distribution of metabolites in a cluster over the eight metabolite classes.

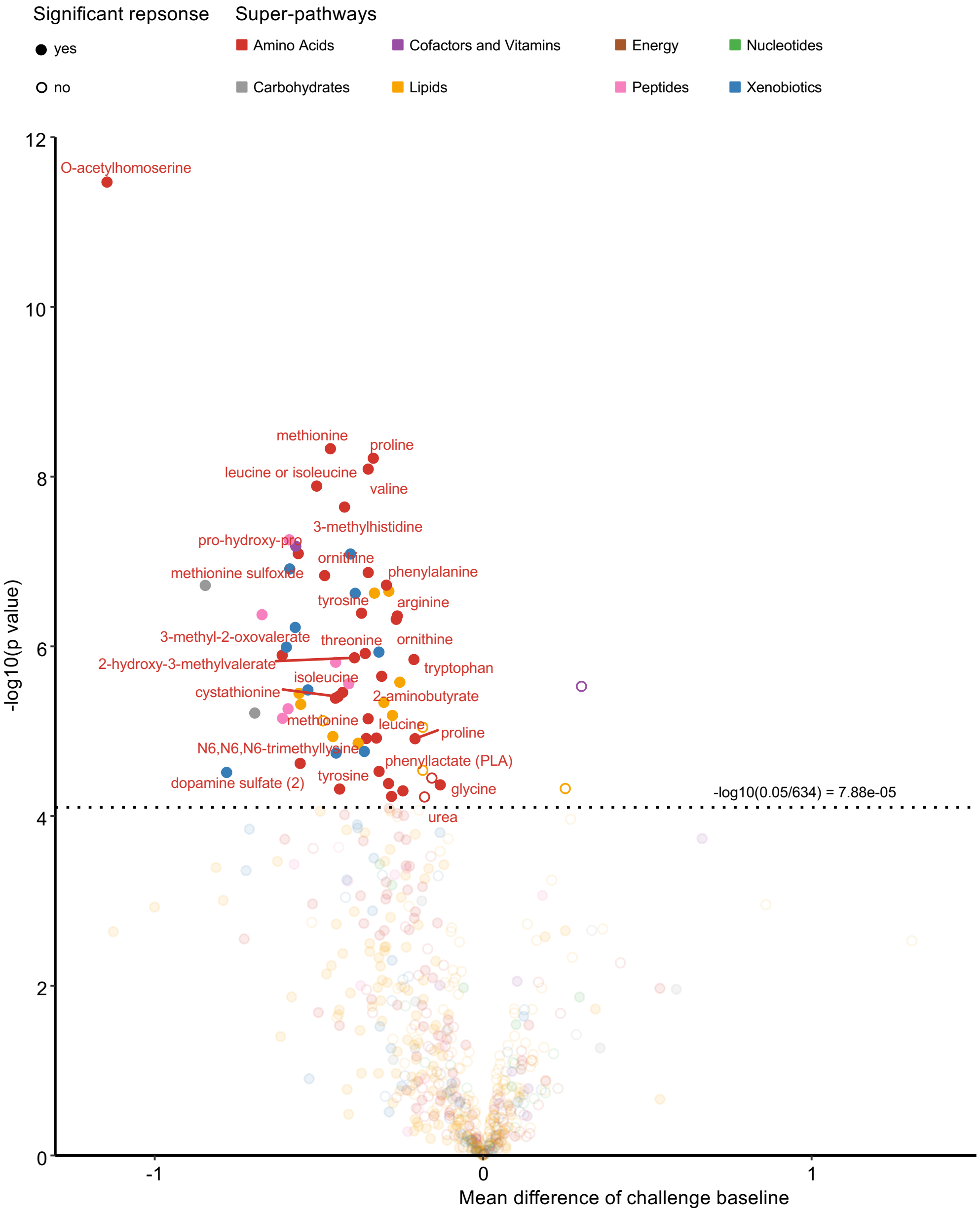

Supplementary Figure 4. Volcano plot displaying differences between challenge baselines. To estimate the magnitude of carry-over effect from OGTT into the SLD challenge, we applied pairwise t-test on the log2 transformed imputed data between OGTT and SLD baseline measurements. The dotted line represents the Bonferroni adjusted significance threshold for P-values at 5% (0.05/634 = 0.000078). The type of circle depicts if metabolites were significant in at least one challenge (full circle) or not (empty circle). Metabolites are colored by super-pathways. The analysis revealed 67 metabolites with significant differences between OGTT and SLD baseline. Metabolites of the 'Amino Acids' class were most affected.
